# Effect of activated carbon/graphite on enhancing anaerobic digestion of waste activated sludge

**DOI:** 10.3389/fmicb.2022.999647

**Published:** 2022-11-15

**Authors:** Fei Wu, Jiaqian Xie, Xiaodong Xin, Junguo He

**Affiliations:** ^1^School of Water, Energy and Environment (SWEE), Cranfield University, Cranfield, United Kingdom; ^2^Department of Environmental Science and Engineering, Huaqiao University, Xiamen, China; ^3^Research Center for Eco-Environmental Engineering, Dongguan University of Technology, Dongguan, China; ^4^School of Civil Engineering, Guangzhou University, Guangzhou, China

**Keywords:** conductive media, activated carbon, graphite, hydrolytic acidification, anaerobic digestion

## Abstract

The conductive media was capable to enhance anaerobic digestion and promote direct interspecific electron transfer (DIET). In this study, the effects of activated carbon- and graphite-conductive media on promoting anaerobic digestion efficiency of waste activated sludge were experimentally studied. The results show that the 100 mesh-activated carbon group reactor produced a largest biogas yield of 468.2 mL/g VSS, which was 13.8% higher than the blank test. The graphite group reactor with 400-grain size produced a largest biogas yield of 462.9 mL/g VSS, which was 12.5% higher than the blank test. Moreover, the optimal particle size of such two carbon- conductive mediators were optimized for enhancing degradation efficiency of VSS, TCOD, total protein and total polysaccharide of waste sludge. Activated carbon was capable to promote the hydrolytic acidification stage in anaerobic digestion of waste sludge. When the particle size reduced to the optimal particle size, the promoting effect could be strengthened for producing more hydrolytic acidification products for methanogenesis. However, in the graphite group, the methane production is increased by promoting the consumption of hydrolysis and acidification products and is enhanced with the particle size reduction, thus promoting the methanogenesis process, and improving the anaerobic digestion efficiency. Microbial community analysis showed that both activated carbon and graphite cultivated the genera of *Methanosaeta*, *Methanobacterium*, *Nitrososphaeraceae*, which promoted the improvement of methane production through the acetate debris methanogenesis pathway.

## Introduction

As a by-product of wastewater biological treatment, the output of waste activated sludge increases obviously in recent years. Anaerobic digestion is one of the most effective and promising treatment technologies to achieve sludge reduction and resource utilization. However, there were still some problems restricted its practical application, such as long residence time, low organic matter degradation rate and gas production efficiency (Geng et al., 2016). Previous research shows that adding external conductive media into the anaerobic digestion process of waste activated sludge would promote the direct interspecies electron transfer (DIET), which is conducive to improve the anaerobic digestion efficiency of waste sludge for completing better methanogenesis (Viggi et al., 2014; Dang et al., 2016; Park et al., 2018; Kassab et al., 2020).

Due to the high specific surface area and excellent conductivity, carbon-based conductive media can promote direct electron transfer between microbial species, thus improving anaerobic digestion efficiency (Lu C. et al., 2020). Peng et al. (2018) showed that the methane yield of waste sludge with activated carbon could be increased by 13% because of its excellent conductivity and high specific surface area. Thus the efficiency of anaerobic digestion with methane production could be boosted clearly. Shrestha et al. (2014) studied the correlation between the microbial flora in the anaerobic digestion system and the conductivity of granular carbon, which found that there was a moderate correlation between the abundance of *geobacterium* spp. in UASB reactor and the conductivity of granular carbon. Such finding indicated that the *Geobacillus* could provide electrons to the main methanogens in anaerobic digestion. Lee et al. (2016) showed that the addition of GAC in the anaerobic digestion process can improve the methane production through direct electron transfer between species, which significantly increased the methane production by 1.8 times. About 34% of the methane formation could be attributed to the attachment of biomass to GAC, indicating that the microbial community through direct electron transfer between species can produce a large proportion of methane. The addition of GAC is beneficial to increase the abundance of microorganisms related to direct electron transfer between species for improving the yield of methane. Yang et al. (2017) added granular activated carbon into the anaerobic digestion reactor, which showed that adding granular activated carbon was conducive to the enrichment of *Geobacillus*, hydrogen consuming methanogens and other electroactive microorganisms. Zhang et al. (2017) added GAC for enhancing methanogenic activities with showing that GAC significantly promoted the generation of methane, while the addition of non-conductive zeolite had no promoting effect. Moreover, the promoting effect is related to the direct interspecific electron transfer between *Geobacteraceae* and *methanosaetaceae*. Although some studies paid much attention on conductive materials (especially the carbon-based ones) for enhancing anaerobic digestion of waste sludge, scarce works elucidated the effects of different carbon-based conductive media on the improvement of anaerobic digestion of waste sludge.

Moreover microbial attachment is affected by the supported properties of the material. Bacteria preferably adhere to moderately rough surfaces with holes a few tenths of microns larger than polished and rough surfaces (Habouzit et al., 2011), along with an intrinsic correlation between microbial communities and the conductivity of anaerobic sludge aggregates (Shrestha et al., 2014). It is therefore anticipated that different adhesion of microbial communities to carbon lineage conductive media of different sizes and pore structures, resulting to differentiation of DIET and methane conversion efficiency. The choice and sizes of the different carbon-based conductive drums may affect the biogas and methane production, so the different carbon-based conductive mediators and sizes were used as the study parameters. However, to the authors’ knowledge, there were few related studies, especially regarding the effect of the adhesion of functional microorganisms to different sizes of different carbon materials on anaerobic digestion.

Therefore, the activated carbon and graphite are investigated for strengthening the anaerobic digestion reaction of waste sludge in this study, while the influence of particle size changes on the anaerobic digestion efficiency associated the mechanism are explored as well. The optimization scheme of adding particle size of carbon-based conductive medium is analyzed, which could provide engineering guide for the practical application of conductive medium for strengthening the anaerobic digestion of waste sludge.

## Materials and methods

### Experimental device and process

The waste activated sludge used in the experiment was taken from the dewatered sludge of Yangjiang sewage plant in Guangdong Province, while the inoculated sludge was taken from the anaerobic fermentation tank of a sludge anaerobic treatment plant in Zhongshan City, Guangdong Province. The mixing ratio of waste activated sludge with inoculated sludge was kept at 4:1. The traits of the waste activated sludge were listed as follows: pH of 7.2, VSS of 26.2 g/L and TCOD of 33,238 mg/L.

On the basis of the result of adding dosage optimization of activated carbon and graphite, the influence of the particle size change from different kinds of conductive materials on enhancing the anaerobic digestion process of waste sludge were investigated. Two groups of anaerobic digestion experiments were designed: *ca.* 500 ml of digestive substrate (i.e., waste sludge) was added into two groups of serum bottles. Each group contained 6 serum bottles (each one with an effective volume of 500 ml). Then the 16 mesh (1 mm), 50 mesh (0.33 mm), 100 mesh (0.16 mm), 200 mesh (0.08 mm), and 400 mesh (0.04 mm) activated carbon and graphite with the corresponding optimal dosage were added into such two groups (i.e., activated carbon and graphite group) of serum bottles, respectively. Among them, take no added conductive material as a blank test. The nitrogen is filled for 10 min to remove oxygen. Then each serum bottle was connected with a biogas for biogas collection and detection. The anaerobic digestion time was set as 25 days. During the experiment, each serum bottle was placed in a constant temperature incubator with a constant temperature of 35°C and an oscillation rate of 120 rpm.

### Analysis method

The pH of sludge was measured by a glass electrode detector (Hanna, Italy). Conductivity is measured by a conductivity meter (Thunder magnetic, DDS-307A).The analysis methods of TSS, VSS and SCOD were carried out according to the standard method (Rice et al., 2012). The concentration and composition of VFAs were measured by Tianmei GC7980 (Shanghai, China) and flame ionization detector. The chromatographic conditions were as follows: injector temperature of 250°C, column temperature of 240°C, oven temperature increased to 170°C with a gradient of 20°C for 2 min, carrier gas of N_2_ (50 ml/min) and H_2_ (55 ml/min; She et al., 2021). The surface morphology of WAS attached to activated carbon and graphite before and after anaerobic digestion was obtained by a Field Emission Scanning Electron Microscopy (ZEISS, Germany). As previously mentioned (She et al., 2021), the digestate samples were collected after sedimentation. The microbial community was measured by Illumina Miseq sequencing technique, following the procedures of DNA extraction, polymerase chain reaction (PCR), quantitative mixing and Miseq sequencing. Firstly, the genomic DNA of sludge microbes was extracted using a E.Z.N.A.^®^ soil DNA Kit (Omega Bio-tek, United States) and checked on 1% agarose gel. The DNA concentration and purity were detected by a spectrophotometer (Thermo Scientific NanoDrop 2000, United States). Then, PCR was conducted through denaturation, annealing and extension processes by a PCR thermocycler (ABI GeneAmp 9700, United States), with 515FmodF (5′-GTGYCAGCMGCCGCGGTAA-3′) and 806RmodR (5′-GGACTACNVGGGTWTCTAAT-3′) which targeted the V4 region of both bacteria and archaea served as primers. After purification, the amplicons were sequenced by the Illumina MiSeq PE300 platform (Illumina, United States), while the raw sequencing data were clustered by 97% similarity to formulate a series of operational taxonomic units (OTUs).

The cumulative methane data were modeled using a modified Gompertz model, as described below (Kafle and Kim, 2013).


(1) $$M=P\cdot \exp\left\{-\exp\left[\frac{R_{\max}\cdot e}{P}\left(\delta -t\right)+1\right]\right\}$$

Where *M* is the cumulative methane production (ml/g VSS), *P* is the production potential (ml/g VSS), *R_max_* is the maximum methane production rate (ml/(g VSS·d)), *δ* is the lag phase (days), *t* is time (days), and *e* = 2.7183.

### Statistical analysis

Origin 8.5 and SPSS 20.0 software were used for statistical analysis. All results were the arithmetic mean of three parallel tests. The standard deviation (±) is represented by origin 8.5 by the estimator with the highest reliability in describing the statistical process. SPSS 20.0 statistical software was used for analysis of variance (ANOVA). One way ANOVA was used to evaluate the significant difference test between samples, and the confidence interval was 95%.

## Results and discussion

### Effect on gas production

#### Daily biogas output

**Figure 1:**
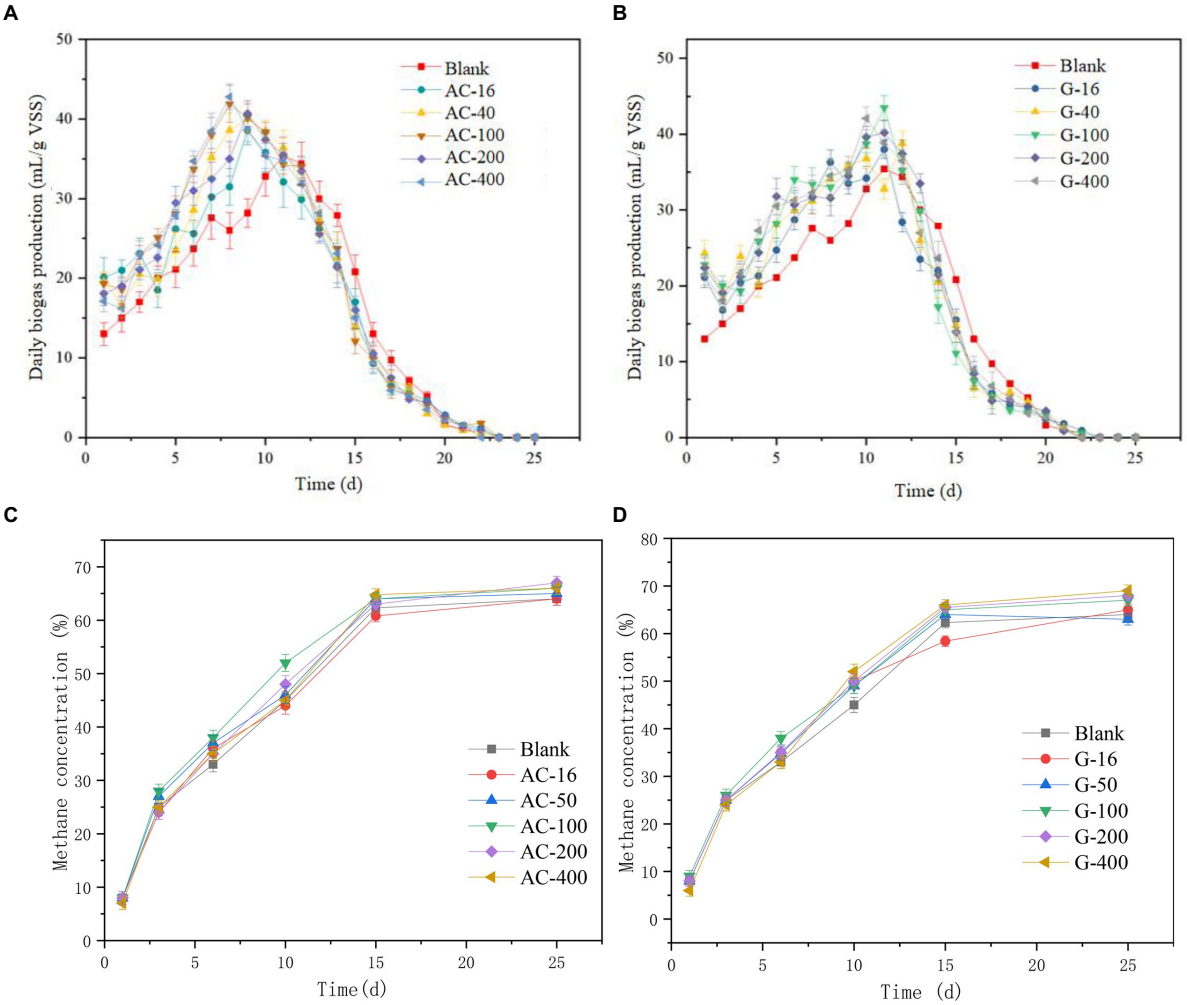
The change of gas production index. **(A)** Changes in daily biogas production of activated carbon reactor; **(B)** Changes in daily biogas production of graphite reactor; **(C)** Change of daily methane production in activated carbon reactor; **(D)** Change of daily methane production in graphite reactor.

The daily biogas production of the bottles added with activated carbon and graphite was shown in Figures 1A,B. The daily biogas production of the activated carbon group increases rapidly from the first day with a peak value occurrence on the 8th day. The 100 mesh- and 400 mesh-bottles reach the peak biogas production on the eighth day (41.9 ml/g VSS) with a daily gas production efficiency of 42.8 ml/g VSS. The 16 mesh-, 50 mesh-, and 200 mesh-bottles reach the peak biogas production efficiency of 31.5, 38.6, and 35.0 ml/g VSS, respectively. This point indicated that the addition of activated carbon can improve the biogas production in the early and middle stages of anaerobic digestion.

The maximum daily biogas production in the graphite group appeared around the 11th day. The 400 mesh-bottle reached a peak value of 42.1 ml/g VSS. The 16 mesh-, 100 mesh-, and 200 mesh-bottles reach the peak biogas production efficiency of 38.0, 43.5, and 40.2 ml/g VSS, respectively. Given the changes of SCOD and VFAs, it can be seen that the activated carbon in the early stage presented a promotion effect on hydrolytic acidification, which boosts the emergence of biogas production. The degradation rate of SCOD and VFAs in graphite group showed that the output of SCOD and VFAs directly affects the methane production stage in anaerobic fermentation, which was consistent with the research results of Lu P. et al. (2020).

The change of methane content (Figures 1C,D) in each bottle presented similar production level. With the progress of anaerobic digestion, the methane content increases rapidly. The methane content in the blank group reactor reached 62.3%, 60.8%~64.8% in the activated carbon group and 58.4%~66.1% in the graphite group, respectively, on 15th day. The change of methane content tends to be stable after the 15th day of the reaction. In general, the addition of activated carbon and graphite can increase the output of biogas and methane. This phenomenon can be attributed to the fact that activated carbon and graphite were conducive to the attachment of microorganisms, thereby enhancing the growth and proliferation of methanogens and promoting electron transfer. Similar phenomena have been found in other studies using various conductive materials (such as biochar, iron nanoparticles, stainless steel and carbon nanotubes) to promote diet in anaerobic digestion (Viggi et al., 2017; Miao et al., 2018; Park et al., 2018; Shanmugam et al., 2018).

#### Change of cumulative biogas production

**Figure 2:**
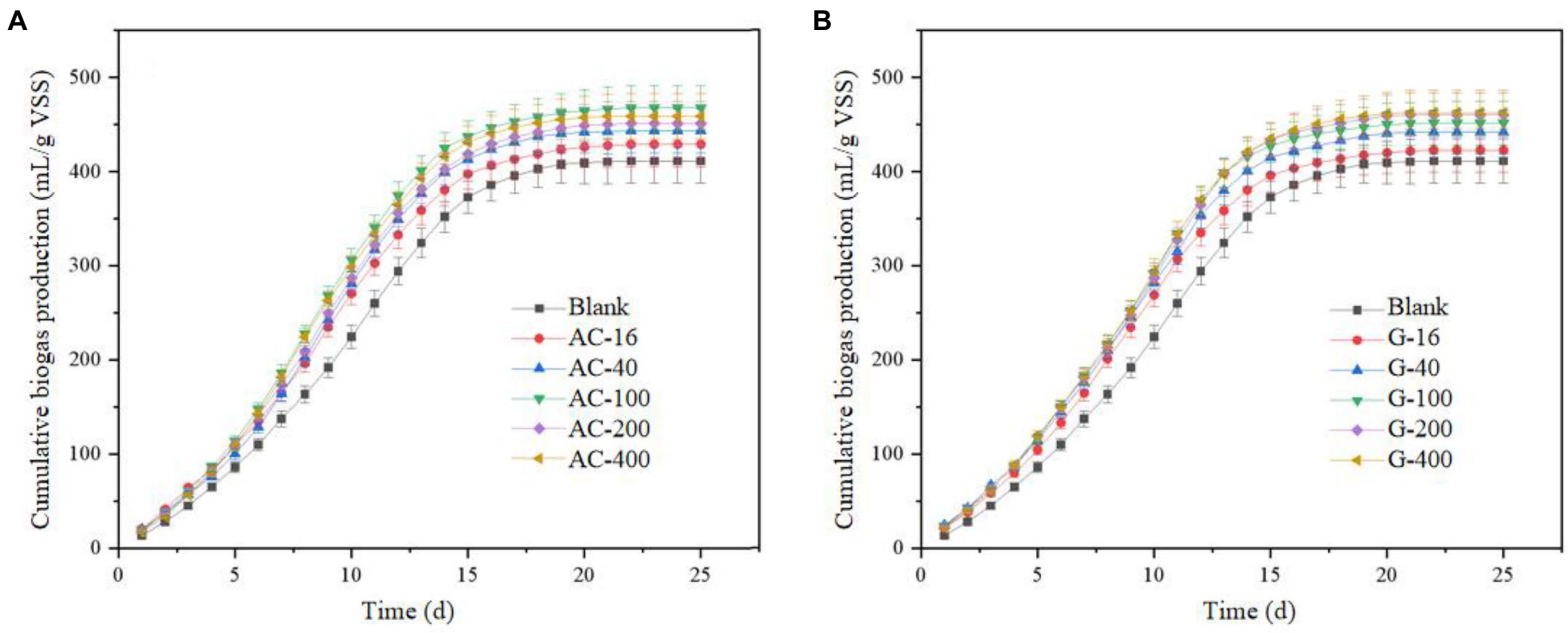
The change of cumulative gas production. **(A)** Activated carbon group; **(B)** Graphite group.

As shown in Figure 2, the total biogas production with activated carbon and graphite addition was increased dramatically *via* anaerobic digestion of waste sludge. The cumulative biogas production efficiency in the blank group was 411.3 ml/g VSS, while the 16 mesh-, 50 mesh-, 100 mesh-, 200 mesh-, and 400 mesh-bottles in activated carbon groups arrived to 429.0, 443.4, 468.2, 451.2, and 459.0 ml/g VSS respectively, which was 4.3%, 7.8%, 13.8%, 9.7%, and 11.6% higher than that of the blank group. The result indicated that the biogas production was improved as the particle size of activated carbon reduced from 16 mesh to 100 mesh. The possible reasons for the increase of the total biogas production in the activated carbon group were as follows: (i) the specific surface area of activated carbon increases with the particle size decrease, while the activated carbon could contact with sludge and microorganisms for enhancing the mass transfer efficiency; (ii) the conductive particles with small particle size were more evenly distributed in the sludge anaerobic digestion system, which was not easy to produce agglomeration inhibition. However, the promotion effect was weakened when the particle size reduces to 400 mesh. The smaller particle size of activated carbon caused a stronger adsorption with resulting in the adsorption of some organic substances, hard to be degraded and bio-transformed.

In the graphite group, the cumulative biogas production of 16, 50, 100, 200, and 400 meshes arrived to 422.9, 442.0, 451.7, 460.2, and 462.9 ml/g VSS, respectively, which was 2.8%, 7.7%, 9.8%, 11.9%, and 12.5% higher than that of the blank group. The change of graphite particle size from 16 mesh to 200 mesh has a significant effect on increasing the total biogas production, while the further improvement effect was not significant when it continues to decrease to 400 mesh. It indicates that the promotion effect on biogas production does not change much with the graphite particle size reduce. The decrease of graphite particle size can contribute to the mass transfer effect for methane bioconversion. The large particles could be easy to be settled and agglomerated with resulting in insufficient contact between sludge and conductive particles, and impairing the methane production efficiency.

#### Cumulative methane production

**Figure 3:**
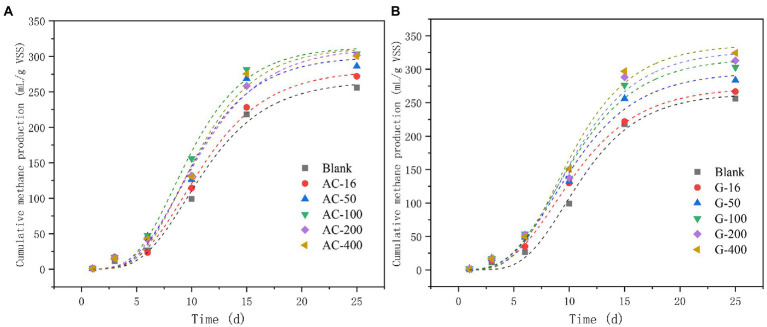
Cumulative methane production. **(A)** Activated carbon group; **(B)** Graphite group.

The changes in cumulative methane production from activated carbon and graphite reactors were shown in Figure 3. The maximum cumulative methane production in 100 mesh-activated carbon and 400 mesh-graphite reactors reached 303.6 mL/g VSS and 324.3 mL/g VSS, which were 1.18% and 1.26% higher than the blank test, respectively. In addition, the coefficients of fitting the cumulative methane production according to the modified Gompertz model were listed in (Table 1). Methane production potential and maximum methane production have increased with the addition of activated carbon and graphite. Among them, the 100 mesh-activated carbon reactor produced a maximum methane potential of 313.66 ± 13.87 mL/g VSS with a maximum methane yield of 32.42 ± 4.25 mL/(g VSS d). The 400 mesh-graphite reactor generated a maximum methane potential of 337.01 ± 18.21 mL/g VSS with a maximum methane yield of 33.28 ± 5.13 mL/(g VSS d). Furthermore, the duration of the lag period can be used to assess the efficiency of anaerobic digestion (Kafle and Kim, 2013). The lag time of the graphite 400 mesh reactor (4.53 days) was shorter than that of the activated carbon 100 mesh reactor (4.82 days), indicating that the fermentation process of the graphite 400 starts faster. Usually the addition of external material may cause prolonged lag periods, as microorganisms require some time to adapt to environmental changes (Lopez-Maury et al., 2008). The positive effect of graphite on shortening the lag time of an anaerobic fermentation system may be attributed to its conductive properties. Mobile graphite can provide additional fast channels for long-distance electron transfer. At the same time, graphite may serve as a supporting material to promote the immobilization of biomass and the development of the microbial community, thus supporting the initiation of methanogenesis in anaerobic fermentation systems (Romero-Gueiza et al., 2016).

**Table 1:** Modified Gompertz model to estimate the parameters.

Samples	P	R_max_	*δ*	R^2^
Blank	265.66 ± 9.21	24.62 ± 0	5.49 ± 0	0.99391
AC-16	281.07 ± 9.19	25.42 ± 0	5.22 ± 0	0.99459
AC-50	299.93 ± 21.82	29.64 ± 6.15	5.08 ± 0.99	0.98537
AC-100	313.66 ± 13.87	32.42 ± 4.25	4.82 ± 0.6	0.99422
AC-200	312.48 ± 13.41	28.19 ± 3.18	4.94 ± 0.57	0.99539
AC-400	312.82 ± 19.36	31.46 ± 5.66	5.32 ± 0.85	0.98944
G-16	272.74 ± 7.61	24.69 ± 1.82	4.67 ± 0.37	0.99799
G-50	295.83 ± 16.73	26.83 ± 4.01	4.65 ± 0.76	0.99156
G-100	317.17 ± 20.38	28.73 ± 4.87	4.62 ± 0.86	0.98922
G-200	327.90 ± 22.90	30.78 ± 5.87	4.87 ± 0.95	0.98703
G-400	337.01 ± 18.21	33.28 ± 5.13	4.53 ± 0.73	0.99188

### Effect of carbon-based conductive materials on pH and dissolved organic matter

#### pH change

**Figure 4:**
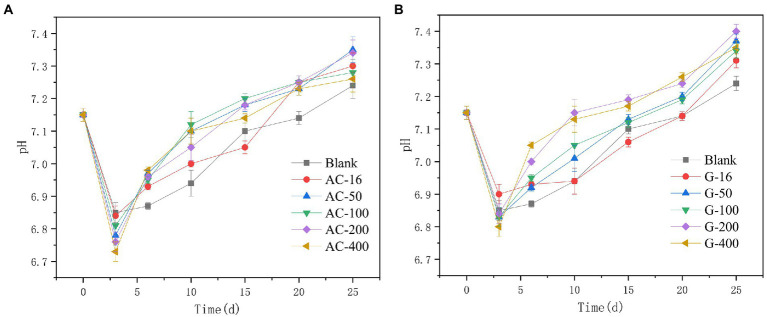
The changes of pH. **(A)** Activated carbon group; **(B)** Graphite group.

It can be seen from Figure 4 that during the anaerobic digestion process, the pH of the anaerobic digestion system decreases in the first 3-days and then increases clearly. At the initial stage, hydrolysis acidification reaction was the main rate-limiting step. The accumulation of volatile fatty acids (VFAs) leads to the decrease of pH. Afterward, the pH in the anaerobic digestion system gradually increases with the rising of the methanogenesis efficiency. The pH in activated carbon group was lower than that of the blank bottle. On 3rd day, the pH of the blank bottle was 6.85, while the pH of the 16 mesh-, 50 mesh-, 100 mesh-, 200 mesh-, and 400 mesh-bottles with activated carbon addition was 6.84, 6.78, 6.81, 6.76, and 6.73, respectively, which indicated that the addition of activated carbon can promote the hydrolysis acidification reaction in the early stage of anaerobic digestion. However, after the 5th day, the pH of the activated carbon group was generally higher than that of the blank, which inferred that the addition of activated carbon can promote the consumption of hydrolytic acidification products and maintain a more suitable pH for methane production. However, the pH in graphite group in the early stage of anaerobic digestion was not significantly different from that in the blank group. The promotion effect on the hydrolysis acidification of anaerobic digestion was not obvious. Subsequently, the pH of other tests seems to be higher than that in the blank bottle, with indicating that the 50 mesh, 100 mesh, 200 mesh, and 400 mesh graphite can promote the consumption of hydrolytic acidification products. The decrease of pH indicates that anaerobic fermentation enters the acid production stage (mainly the production of VFAs). The gradual increase of pH means that VFAs were consumed and utilized as methanogenic substrates, which was consistent with the research phenomenon of Chen et al. (2022) with stating that pH of 7.0~9.0 was conducive to the production of methane (Zhao et al., 2021).

#### Change of SCOD concentration

**Figure 5:**
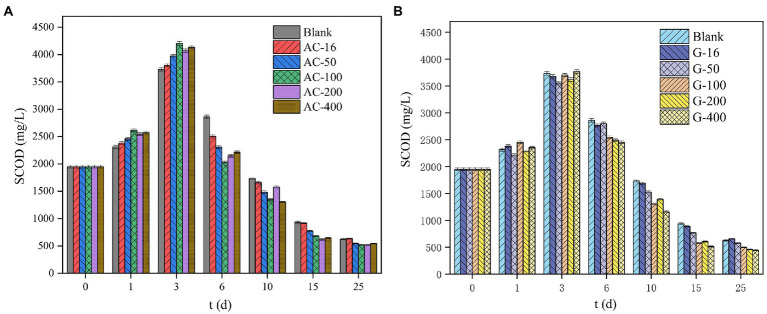
The change of SCOD concentration. **(A)** Activated carbon reactor; **(B)** Graphite reactor.

During the initial stage of anaerobic digestion, the hydrolysis and acidification emerged dominantly. Various complex macromolecular organics such as polysaccharides and proteins could be decomposed into small molecule soluble organics under the action of hydrolysis acidification bacteria, which provided sufficient substrates for the subsequent methanogenesis stage (Zhao et al., 2021). Figure 5 depicted the changes of SCOD concentration in the blank group, activated carbon and graphite groups during anaerobic digestion of waste sludge. The SCOD concentration in each bottle increased rapidly in the first 3 days (Figures 2A,B). During anaerobic fermentation, hydrolysis was considered as the rate-limiting step, which determines the fermentation efficiency of the sludge (Ge et al., 2011). The SCOD concentration of 16 mesh, 50 mesh, 100 mesh, 200 mesh, and 400 mesh reactors in the activated carbon group arrived to 3,805, 3,973, 4,205, 4,073, and 4,135 mg/l respectively, which was increased by 2.0%, 6.5%, 12.6%, 9.1%, and 10.8% respectively, as compared with that in the blank group. The increase of SCOD indicates that the addition of graphite promotes the hydrolysis process and transferring the sludge organic matter from the solid phase to the liquid phase (Hu et al., 2011). This point indicated that the addition of activated carbon with different particle sizes presented different promotion effect on boosting the hydrolysis stage. Overall, adding activated carbon particles with smaller particle size in the range of 16 mesh to 100 mesh has a better boosting effect on the hydrolysis reaction. But when the particle size was further reduced to 400 mesh, the promotion effect was weakened. Microbial adhesion was governed by the adsorption capacity of the material, which in turn depends on the surface morphology, pore volume and size, hydrophobicity, and ion exchange capacity (Kubota et al., 2008; Masebinu et al., 2019). It was possible that at the same dosage, the activated carbon with small particle size provides a larger surface area that the organisms can attach, which was more favorable for anaerobic digestion to proceed in the direction of hydrolysis. When the particle size was too small, the organisms were not easy to adhere to the surface, easy to be washed away. On the 6th day, the SCOD concentration in each reactor of activated carbon group was significantly lower than that in the graphite group. On the one hand, the conductivity of activated carbon promotes the methanogenesis process, which speeds up the consumption of hydrolytic acidification products. On the other hand, the adsorption performance of activated carbon leads to the adsorption of partial SCOD, consistent with the findings of the previous studies (Watanabe, 2008; Pham et al., 2009).

However, the SCOD changes in graphite group was similar with that in the blank group. After the 3rd day, the SCOD concentration began to decrease gradually, caused by the continuous consumption of dissolved organic matter for methanogenesis. On the 6th day, the SCOD in the 16 mesh, 50 mesh, 100 mesh, 200 mesh, and 400 mesh graphite bottles arrived to 2,767, 2,801, 2,545, 2,489, and 2,453 mg/l respectively, which was 3.3%, 2.1%, 11.1%, 13.0%, and 14.3% lower than that in the blank group. Such results showed that adding graphite with smaller particle size had a better effect on promoting anaerobic digestion.

#### Change of VFAs concentration

**Figure 6:**
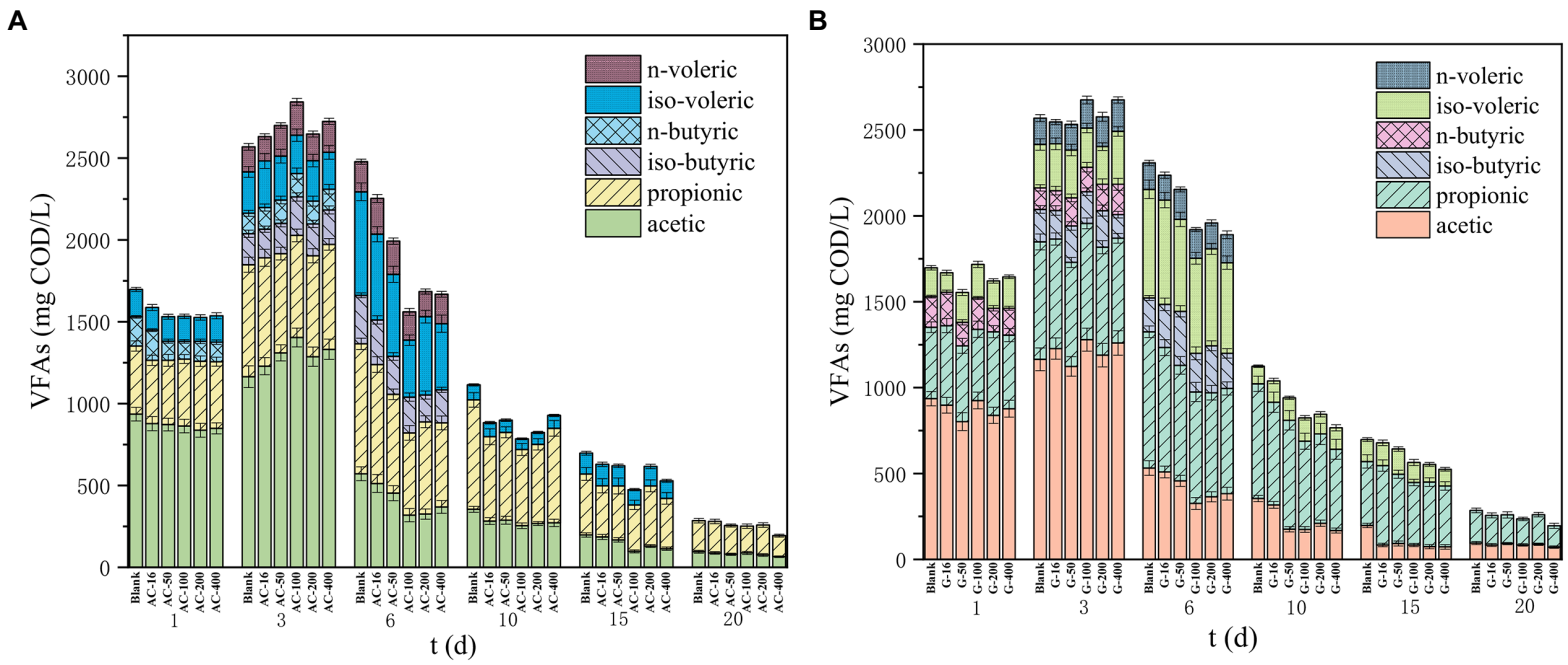
The change of VFAs concentration. **(A)** Activated carbon reactor; **(B)** Graphite reactor.

VFAs were the intermediate products during waste sludge anaerobic digestion process. The concentration of VFAs can reflect the efficiency of sludge hydrolysis and acidification (She et al., 2020). From Figure 6, the VFAs in each group gradually increased in the first 3 days. Finally, VFAs in the blank group was 2,569 mg/l, while the VFAs in the 16 mesh-, 50 mesh-, 100 mesh-, 200 mesh-, and 400 mesh- bottle with the activated carbon addition reached 2,632, 2,699, 2,843, 2,647, and 2,724 mg/l respectively, which was 2.5%, 5.1%, 10.7%, 3.0%, and 6.0% higher than the blank one. This finding indicated that the activated carbon can promote the formation of volatile acids. Moreover, the promotion effect was enhanced when the particle size changes from 16 mesh to 100 mesh. In the AC-100 reactor, the acetic acid was better enriched, promoting the utilization of acetic acid by the acetic acid-producing methanogens and increasing the methane yield.

On the third day, the VFAs concentration in the graphite group did not differ significantly, which suggested that the addition of graphite had little effect on the formation of VFAs. On the 6th day, the degradation rate of VFAs was faster. The VFAs concentration in the blank group was 2,308 mg/L, while the VFAs concentration in the 16 mesh-, 50 mesh-, 100 mesh-, and 200 mesh-bottles in graphite group arrived to 2,237, 2,154, 1920, 1959, and 1890 mg/l, respectively, which was 3.1%, 6.7%, 16.8%, 15.1%, and 18.1% lower than that in the blank group. Adding graphite can accelerate the consumption of hydrolytic acidification products, thus significantly promoting the methanogenesis process (Lu C et al., 2020; Xie et al., 2020). It was reported that the proportion of main products in glucose acidogenesis (acetate, propionate, butyrate, etc) was affected by various operating factors such as pH, temperature, and hydraulic retention time (Xu et al., 2014). The acetate can be generated from the oxidation of propionate and butyrate, according to Eqs. (2–3), while the methane production mainly depended upon hydrogen nutrition and acetate nutrition (Abbasi et al., 2012).


(2) $$CH_{3}CH_{2}CH_{2}COO^{-}+2H_{2}O\to 2CH_{3}COO^{-}+H^{+}+2H_{2}$$

(3) $$CH_{3}CH_{2}COO^{-}+2H_{2}O\to CH_{3}COO^{-}+CO_{2}+3H_{2}$$

### Effect on sludge reduction

**Figure 7:**
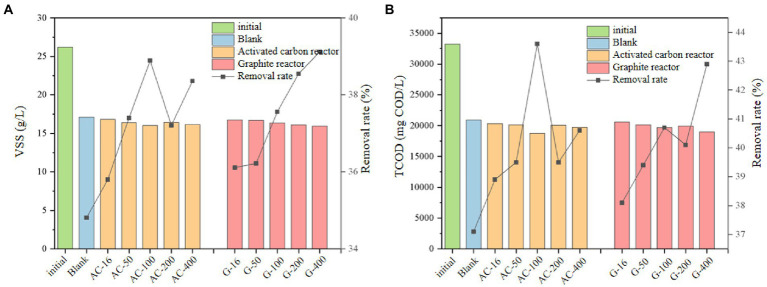
The change of total organic matter concentration. **(A)** VSS; **(B)** TCOD.

The changes of VSS and TCOD concentrations in the blank bottle, activated carbon and graphite group before and after anaerobic digestion were shown in Figure 7. From Figure 7A, the VSS in activated carbon group was lower than that in the blank group. The VSS removal efficiency of the blank group was 34.8%, while it increased from 35.8% to 38.9% in the activated carbon group as the particle size decreased from 16 mesh to 100 mesh. It changed to 38.4% as the particle size continued to increase from 100 mesh to 400 mesh. In the graphite group, the VSS removal efficiency increased from 36.1% to 39.1% with the increase of graphite particle size from 16 mesh to 400 mesh, which indicated that adding graphite particles with smaller particle size was conducive to the removal of VSS, which promotes the transfer of sludge organic matter from solid phase to liquid phase, which conforms to the rising law of SCOD (Hu et al., 2011; Wu et al., 2019).

The changes of TCOD (Figure 7B) before and after anaerobic digestion seems to be consistent with the shifts of VSS. The TCOD in the activated carbon and graphite group present a lower level than that in the blank test. As the particle size was reduced from 16 mesh to 100 mesh, the removal efficiency of TCOD in the activated carbon group increases from 38.9% to 43.6%. As the particle size continued to increase from 100 mesh to 400 mesh, the removal efficiency of TCOD decreased to 40.6%. Moreover, the increase of graphite particle size from 16 mesh to 400 mesh leads to an increasing removal efficiency of TCOD from 38.1% to 42.9%. It can be seen that reducing the particle size of graphite contributes to the improvement of sludge reduction.

### Effect on conductivity of sludge system

**Figure 8:**
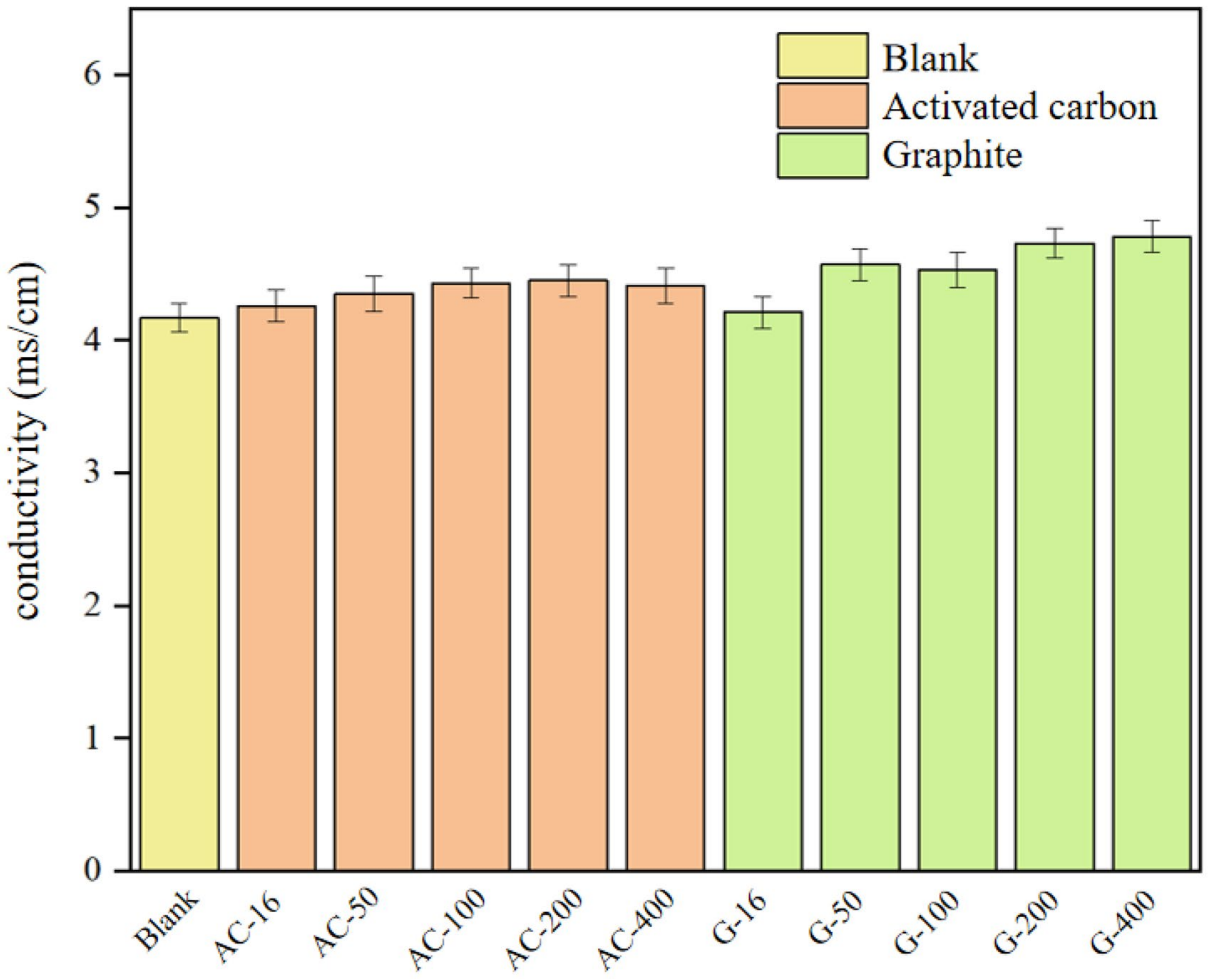
The conductivity of sludge supernatant.

The conductivity of the sludge supernatant of each carbon-adding series bottle was shown in Figure 8. After adding activated carbon, the conductivity of the sludge supernatant does not change significantly as compared with that of the blank test. The highest conductivity of the activated carbon group was 4.39 ms/cm at the 200 mesh-bottle, which was only 5.3% higher than that of the blank test. The conductivity in the graphite group was higher than that in the blank test, such as the 4.70 ms/cm in the 400 mesh graphite bottle, which was 12.7% higher than that in the blank test. The higher conductivity of graphite group than that in activated carbon group may be caused by the better conductivity of graphite. The conductivity was higher when the particle size of the graphite group was 200~400 mesh than the system with the particle size of 16~100 mesh, which indicated that reducing the particle size of the conductive medium can further improve the conductivity of the digestion system and promote the electron transfer efficiency.

### Effect on sludge surface morphology

**Figure 9:**
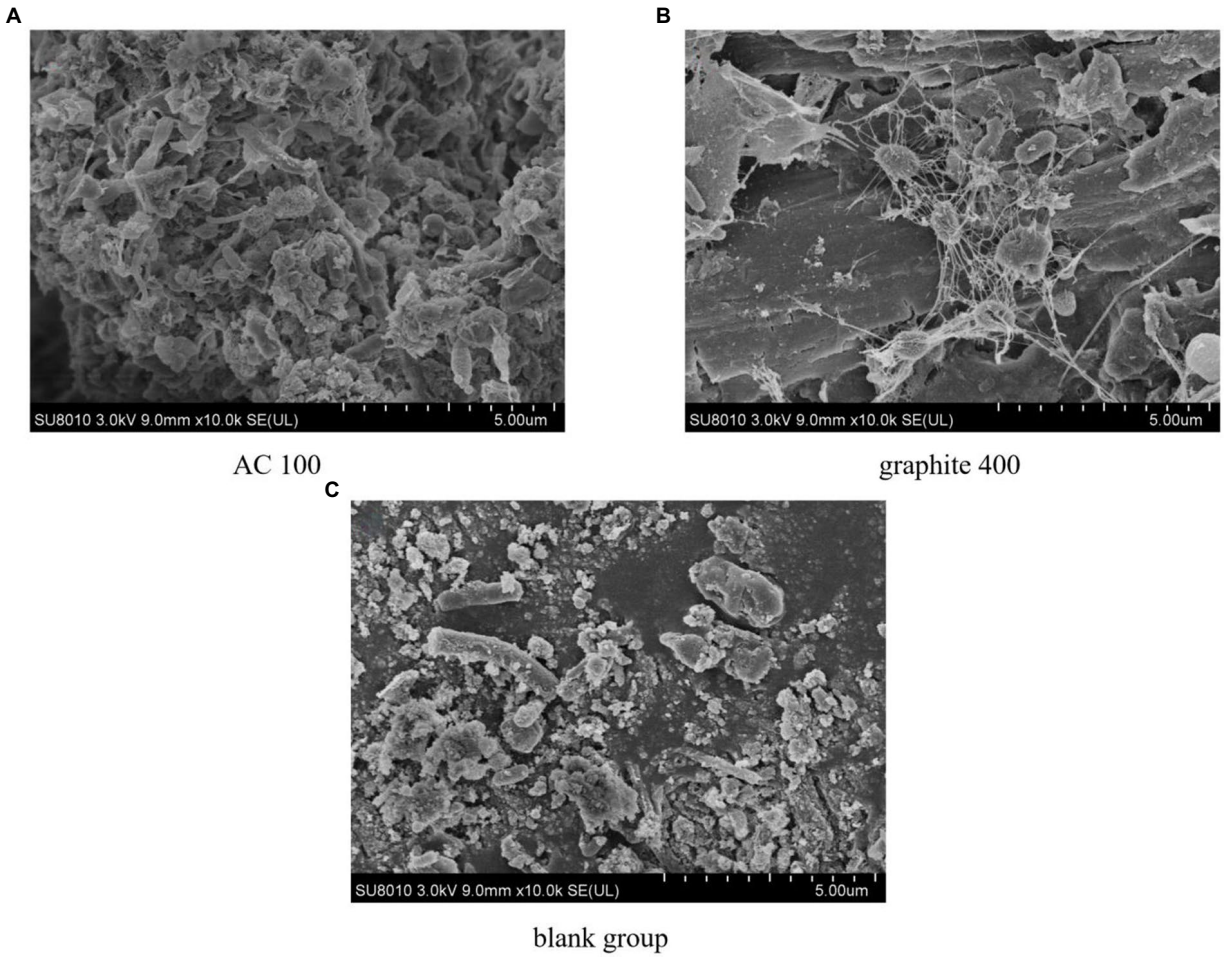
The SEM of sludge. **(A)** AC 100. **(B)** Graphite 400. **(C)** Blank group.

The morphology of the sludge surface after anaerobic digestion reaction in the activated carbon group (100 mesh) was shown in Figure 9A. Attachment was the first step of biofilm formation. If an electrical connection was established between microorganisms and materials, it should be firmly attached to the surface of materials as expected (Johnravindar et al., 2020). Due to the porous structure of the activated carbon, microorganisms were enriched on the surface and pores of the activated carbon. Among them, the dominant bacteria were *bacilli* and *cocci*. The microbial species transfer electrons through activated carbon or direct contact, which greatly improves the efficiency of direct electron transfer (Dinh et al., 2004). The Figure 9B shows the morphology of the sludge surface after anaerobic digestion in the graphite group (400 mesh). The microorganisms were closely attached to the surface of the graphite particles, which contributes to the excellent conductivity of the graphite through direct contact to shorten the electron transfer distance (Viggi et al., 2014); In addition, there were a large number of hyphae connected between microorganisms, which was conducive to interspecific electron transfer through conductive hyphae and more effective interspecific electron transfer between interoperable microorganisms. Microorganisms initially convert complex organic compounds into volatile fatty acids, CO_2_ and H_2_O, which were then converted into methane by methanogenic archaea. The methanogenesis, as a limiting reaction in anaerobic fermentation, was relatively slow (Habouzit et al., 2014). However, the microorganisms attached to the surface of the graphite were not close to those of the activated carbon. This phenomenon will accelerate the mass transfer between microorganisms and result in the biogas production. Such a situation also has been found in the research of Johnravindar et al. (2020).

As shown in Figure 9C, the enrichment of microorganisms in the control group was weaker than that of adding conductive media. Traditional small molecular substances such as hydrogen /formic acid can be used as the carrier of electron transfer between microorganisms, which contributes to a low methane production efficiency (Baek et al., 2018). The microorganisms in the sludge anaerobic digestion with activated carbon and graphite addition were closely attached to the surface of the conductive medium, which was conducive to direct interspecific electron transfer efficiency (Yang et al., 2017).

### Effect on microbial community

**Figure 10:**
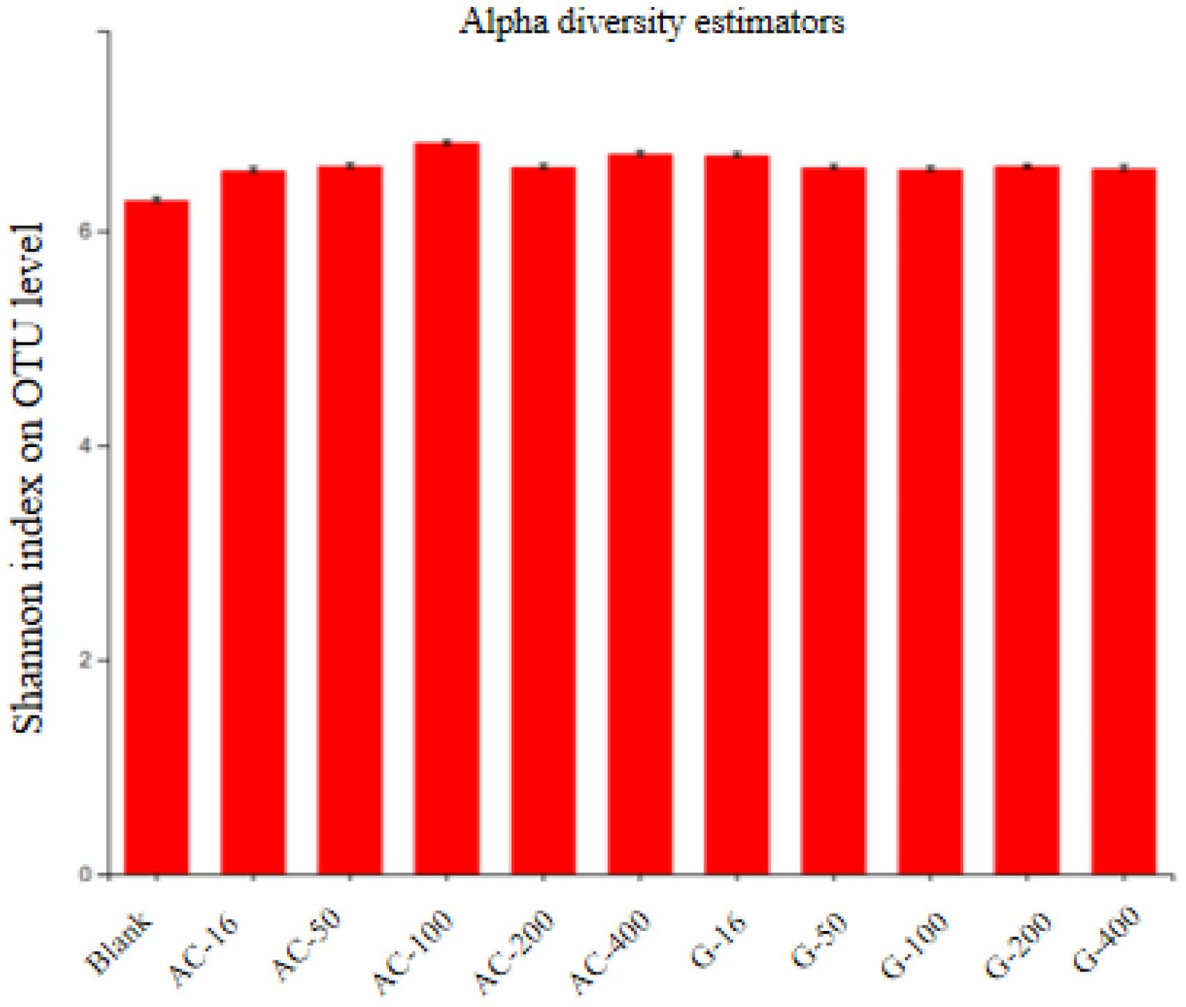
The Shannon index in different reactors.

Figure 10 depicts the microbial diversity after anaerobic digestion of each reactor added with activated carbon and graphite. The Shannon index of each reactor added with activated carbon and graphite was larger than that of the blank group. The Shannon index of the blank group reactor was 6.29, while the Shannon index of the 16 mesh, 50 mesh, 100 mesh, 200 mesh, and 400 mesh bottles in the activated carbon group was 6.56, 6.60, 6.82, 6.59, and 6.72, respectively. As the particle size decreases from 16 mesh to 100 mesh, the Shannon index increases. As the particle size further decrease causing the Shannon index reduction, indicating that the addition of activated carbon can promote the microbial population diversity. The Shannon index of the bottles with different particle sizes of graphite group seems to be similar. It inferred that the promotion effect was less affected by the change of particle size. Activated carbon promotes the improvement of microbial diversity in anaerobic fermentation reactor, makes the microbial community structure more stable, which was more conducive to the utilization of organic matter by microorganisms for methane production (He et al., 2019; Wang et al., 2019).

**Figure 11:**
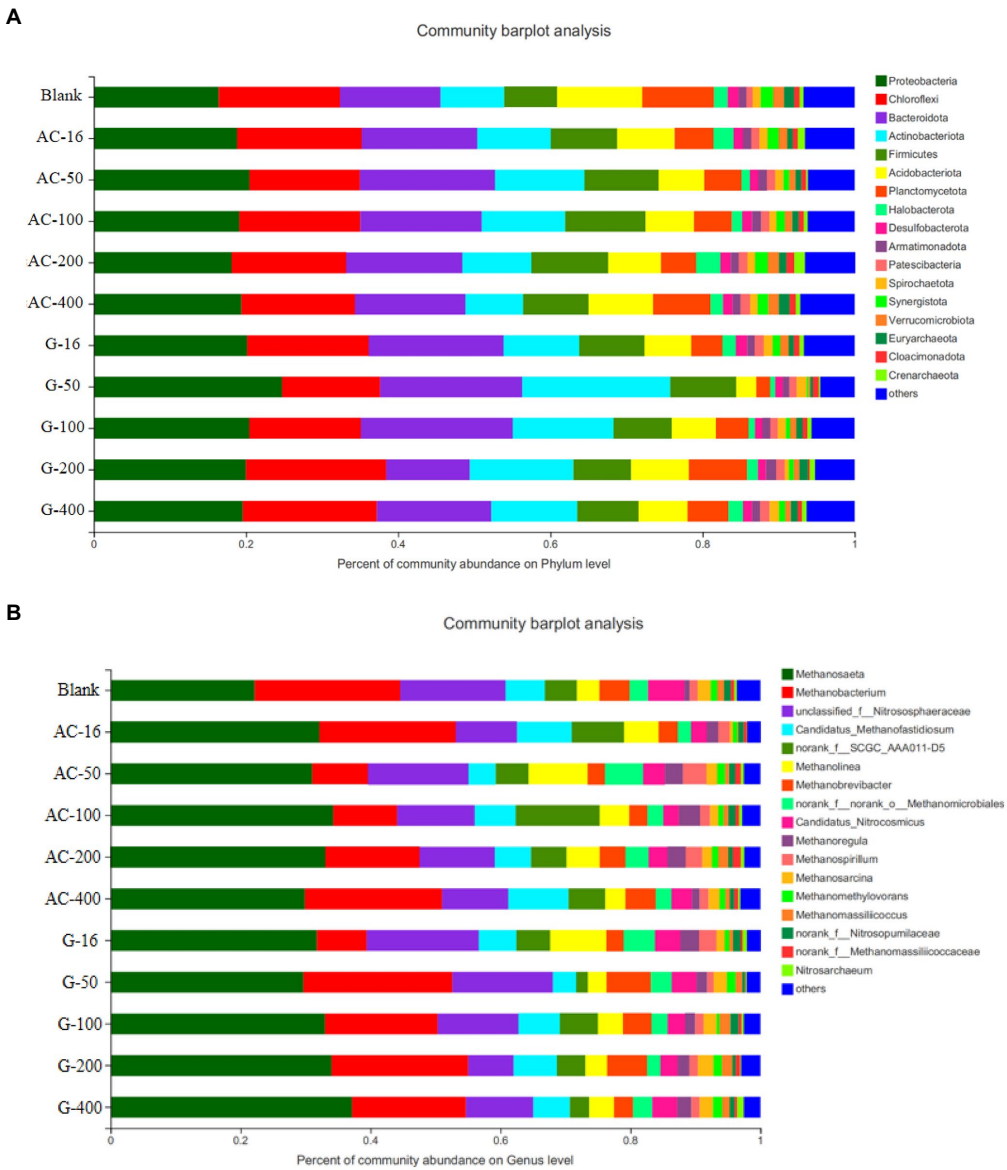
Distribution of microbial community in different reactors. **(A)** Bacterial community structure at phylum level; **(B)** Archaeal community structure at the genus level.

In the community structure at phylum level (Figure 11A), *Firmicutes* were typical interoperating bacteria that degrade different organic matter to produce VFAs, which can accelerate the production of acetate (Shrestha et al., 2014). The abundance of *Firmicutes* in the blank group reactor was 6.9%, while the abundance of *Methanosaeta* in the activated carbon group was 8.7%, 9.8%, 10.6%, 10.0%, and 8.6% in the 16 mesh, 50 mesh, 100 mesh, 200 mesh, and 400 mesh bottles, respectively. The enrichment of *Firmicutes* was conducive to promoting the hydrolysis acidification reaction for the subsequent methanogenesis stage (She et al., 2020).

Figure 11B shows the Archaeal community structure at genus level after anaerobic digestion. The dominant common archaea contain *Methanosaeta*, *Methanobacterium*, *Nitrososphaeraceae* etc. *Methanosaeta* was a typical acetate debris methanogen capable of converting acetate to methane and carbon dioxide (Chen et al., 2017). *Methanobacterium* was a hydrotrophic methanogenic archaeon, which mainly uses hydrogen to reduce carbon dioxide to methane (Maus et al., 2013). *Nitrososphaeraceae* mainly dominates the nitrification effect (Yang et al., 2021). The abundance decreased in both the *Nitrososphaeraceae* activated carbon and graphite reactors, indicating that the genera concerning nitrification were reduced in the reactor, and the methanogenic-related genera were enriched in the reactor. The abundance of *Methanosaeta* in the blank test was 22.1%, while the abundance of *Methanosaeta* in the activated carbon group was 32.1%, 31.0%, 34.2%, 33.1%, and 29.9% in the 16 mesh, 50 mesh, 100 mesh, 200 mesh, and 400 mesh bottles, respectively, which was significantly higher than that in the blank test. The enrichment of *Methanosaeta* methanogens, an electrically active microorganism, was conducive to promoting the direct electron transfer between species (Chen et al., 2017). It can obviously promote the efficiency of methane production. However, in the 16 mesh-, 50 mesh-, 100 mesh-, 200 mesh-, and 400 mesh-bottles of the graphite group, the *Methanosaeta* abundance was 31.7%, 29.7%, 33.0%, 34.0%, and 37.1%, respectively. As the particle size decreases from 16 mesh to 400 mesh, the abundance of *Methanosaeta* increases, indicating that reducing the particle size of graphite can promote the enrichment of methanogenic functional microorganisms for enhancing methanogenic process.

By comparing the abundance of *Methanosaeta* in activated carbon and graphite group, the enrichment in 400 mesh bottle in graphite group was better than that in 100 mesh bottle in activated carbon group. However, the total biogas production of 100 mesh group in activated carbon group was higher than that of 400 mesh group in graphite group, which suggested that activated carbon can contribute to VFAs production. The advantage of *Methanosaeta* indicates that acetic acid fragmentation was the main methanogenic pathway (shown in Figure 11B). A few *Methanosaeta* species have been demonstrated to carry out DIET in the presence of conductive materials (Rotaru et al., 2014, 2018). This indicates that activated carbon and graphite materials may promote the formation of acetic acid/ methane, which was consistent with recent studies (Li et al., 2018; Xiao et al., 2019). In addition, the *Methanobacterium* cannot perform DIET, which may be the reason for the reduction of *Methanobacterium* in activated carbon- and graphite-systems (Rotaru et al., 2014). Syntrophic bacteria capable of DIET, such as *Geobacter* and *Syntrophomonas*, were not detected in any digestive tract (Lovley, 2017; Yin and Wu, 2019). Therefore, it was speculated that some unknown DIET-executing bacteria could use activated carbon/graphite as conductivity tubes to establish extracellular electron transfer *via* an association with *Methanosaeta* so as to promote the generation of methane.

## Conclusion

The cumulative biogas production efficiency of 468.2 ml/g VSS with adding 100 mesh particle size of activated carbon was realized through anaerobic digestion of waste sludge, while it changed as 462.9 ml/g VSS with a particle size of 400 mesh in the graphite group. Activated carbon promotes methanogenesis by promoting the hydrolysis process and producing more hydrolyzed acidification products, and graphite promotes methanogenesis by promoting the consumption of the hydrolyzed acidification products. Adding activated carbon and graphite to the anaerobic digestion reactor was conducive to the interspecific electron transmission between the operating microorganisms, and can improve the conductivity of the sludge system, and promote the electron transmission of the system; On the other hand, it enriched the genera of *Methanosaeta*, *Methanobacterium* and *Nitrososphaeraceae*, which promotes methane production through the acetate trophic pathway.

## Data availability statement

The original contributions presented in the study are included in the article/supplementary material, further inquiries can be directed to the corresponding author.

## Author contributions

FW completed the draft manuscript. JX was responsible for the experiment data obtainment with equal contribution. XX conducted the data analysis. JH supervised the manuscript. All authors contributed to the article and approved the submitted version.

## Funding

This study was financially supported by National Natural Science Foundation of China (52070048) and Research and Development Plan in Key Areas of Guangdong Province (2019B110209002).

## Conflict of interest

The authors declare that the research was conducted in the absence of any commercial or financial relationships that could be construed as a potential conflict of interest.

## Publisher’s note

All claims expressed in this article are solely those of the authors and do not necessarily represent those of their affiliated organizations, or those of the publisher, the editors and the reviewers. Any product that may be evaluated in this article, or claim that may be made by its manufacturer, is not guaranteed or endorsed by the publisher.
